# Development of an AI Model to Measure Traffic Air Pollution from Multisensor and Weather Data

**DOI:** 10.3390/s19224941

**Published:** 2019-11-13

**Authors:** Hai-Bang Ly, Lu Minh Le, Luong Van Phi, Viet-Hung Phan, Van Quan Tran, Binh Thai Pham, Tien-Thinh Le, Sybil Derrible

**Affiliations:** 1 University of Transport Technology, Hanoi 100000, Vietnam; banglh@utt.edu.vn (H.-B.L.); vanpl@utt.edu.vn (L.V.P.); quantv@utt.edu.vn (V.Q.T.); 2 Faculty of Engineering, Vietnam National University of Agriculture, Gia Lam, Hanoi 100000, Vietnam; lmlu@vnua.edu.vn; 3 University of Transport and Communications, Ha Noi 100000, Vietnam; phanviethung@utc.edu.vn; 4 Institute of Research and Development, Duy Tan University, Da Nang 550000, Vietnam; 5 Department of Civil and Materials Engineering, Institute of Environmental Science and Policy, University of Illinois at Chicago, Chicago, IL 60607, USA

**Keywords:** air quality, adaptive neuro-fuzzy inference system, particle swarm optimization, simulated annealing

## Abstract

Gas multisensor devices offer an effective approach to monitor air pollution, which has become a pandemic in many cities, especially because of transport emissions. To be reliable, properly trained models need to be developed that combine output from sensors with weather data; however, many factors can affect the accuracy of the models. The main objective of this study was to explore the impact of several input variables in training different air quality indexes using fuzzy logic combined with two metaheuristic optimizations: simulated annealing (SA) and particle swarm optimization (PSO). In this work, the concentrations of NO_2_ and CO were predicted using five resistivities from multisensor devices and three weather variables (temperature, relative humidity, and absolute humidity). In order to validate the results, several measures were calculated, including the correlation coefficient and the mean absolute error. Overall, PSO was found to perform the best. Finally, input resistivities of NO_2_ and nonmetanic hydrocarbons (NMHC) were found to be the most sensitive to predict concentrations of NO_2_ and CO.

## 1. Introduction

In the transport sector, fossil fuel-powered vehicles, such as motorcycles, cars, and buses, are major contributors to local air pollution [1]. Two particularly important compounds in air pollution are nitrogen oxides (NO_x_) and carbon monoxide (CO). On the one hand, primary NO_x_ emissions are mostly in the form of nitric oxide (NO), which can react with ozone (O_3_) to form nitrogen dioxide (NO_2_). On the other hand, CO is produced by an incomplete combustion of fossil fuels, such as gasoline, natural gas, oil, coal, and wood. Emissions from transport vehicles are responsible for more than half of the NO_x_ in the air and represent the largest anthropogenic source of CO [2,3]. In densely populated cities and industrialized areas, air quality has become an important measure of quality of life, as is the case in Vietnam. In fact, many studies have found that pollutants from vehicle exhaust can cause adverse impacts on nearly every organ in the body [4,5,6,7,8,9,10]. Controlling air quality (by controlling air pollution) is highly desirable to improve urban sustainability and quality of life [11], and it starts by measuring and forecasting air quality.

In the literature, two families of techniques are typically used to forecast pollutant concentrations or determine the factors that control NO_2_ and CO concentrations. The first family uses detailed atmospheric diffusion models, which take into account the physical and chemical equations that impact pollutant concentrations [12,13,14,15,16]. The second family applies statistical methods and leverages statistical models to capture the fundamental relationship between a set of input data (i.e., independent variables) and their targets (i.e., dependent variables) [17,18,19,20,21,22,23,24,25]. As an example, Shi and Harrison [26] developed a linear regression model to predict NO_x_ and NO_2_ concentrations in London.

In parallel, low-cost gas multisensor technology can potentially revolutionize the research on air pollution by providing highly disaggregate spatiotemporal pollution data. These data can be utilized to supplement traditional pollution monitoring methods to help improve air pollution estimates and raise awareness about air pollution. Nonetheless, data quality and data processing remain an important concern, which hinders the adoption of these low-cost sensors. Indeed, unreliable sensors can easily provide erroneous data, which may then inform the wrong policies.

To partly address these concerns, artificial intelligence (AI) can offer an effective numerical approach to model complex and nonlinear relationships between a set of input data and targets, and it has been applied to many fields, from transport [27,28] to water resource engineering [29,30]. For air quality, artificial neural networks (ANN) can model nonlinear systems, and they have been successfully used to model sulfur dioxide concentrations in the industrial site of Priolo, Syracuse, Italy [31]. Comrie et al. [32] compared multilayer perceptron (MLP) models with more traditional regression models for ozone forecasting. Focusing on Central London (UK), Gardner and Dorling [33] developed a MLP model with hourly NO_x_ and NO_2_ data as well as meteorological condition data and showed that MLP outperformed the regression models developed by Shi and Harrison [26] using the same study site.

As the relationship between NO_2_, CO, and meteorology is complex and nonlinear, we developed two AI models to predict hourly NO_2_ and CO concentrations from readily observable local meteorological data. The two models were adaptive neuro-fuzzy inference system (ANFIS) optimized by particle swarm optimization (hereafter denoted as ANFIS-PSO) and ANFIS optimized by simulated annealing (hereafter denoted as ANFIS-SA). The main objective of this study was to explore the influence of input data on predicting different air quality indexes. The input parameters were divided into two main groups: (i) resistivities from multisensor devices, which included five inputs, and (ii) meteorological variables, including temperature, relative humidity, and absolute humidity. Furthermore, a sensitivity analysis was performed to determine the most important factors that affect air quality, specifically to identify the dominant links between the sensors and the pollutants. The data was collected in the center of a city in Italy between March 2004 and February 2005.

## 2. Methods Used

### 2.1. Machine Learning Methods

#### 2.1.1. Adaptive Network-Based Fuzzy Inference System

The ANFIS algorithm combines fuzzy systems with neural networks. Jang [34] first proposed the algorithm and used it to investigate nonlinear systems. Generally, an ANFIS includes five layers, and each layer is formulated by some nodes and node functions [35]. In this study, we used the Takagi–Sugeno model, considered to be the most prominent fuzzy inference system model [36,37,38].

#### 2.1.2. Particle Swarm Optimization

Since its introduction by Kennedy and Eberhart [39], PSO has become one of the most commonly used evolutionary methods for parameter optimization. The principle of PSO is based on the social and biological behaviors of animals when seeking food. PSO originates with a random group of particles, where each particle stands for a specific solution to the problem. It comprises groups of particles in which the position of each individual is affected by the position of the particles in the group. Essentially, each individual can adjust their position in the search space based on the best locations possible and the best locations adjacent to their neighbors. At every iteration step, the position of each particle is also updated based on its current position and velocity [40].

Moreover, each particle randomly moves along the search space, but it can get disrupted as a result of its own knowledge and that of its neighbors [41,42]. Therefore, the way a particle searches can be influenced by other particles in the swarm. This means that the particles learn and acquire knowledge from one another in a group and advance at the same rate as their best neighbors [41,42]. Combining regression modeling and PSO generally results in a high-performing model that is suitable for addressing classification and forecasting problems [41,42]. For more information on PSO, the reader is referred to [43,44,45].

#### 2.1.3. Simulated Annealing

Simulated annealing was developed after PSO, and it has become a powerful tool for global optimization. Based on the similarity between a search algorithm and the process of annealing in metallurgy, the idea of simulated annealing first appeared in Metropolis et al. [46] as a simulation algorithm. Similar to a cooling process, the algorithm simulates a steady temperature decrease until the system converges to a stable state, thereby avoiding the inclusion of defects when cooling too quickly or too slowly. Search algorithms also focus on identifying solutions without ignoring better solutions that can be found later. Kirkpatrick et al. and Cerny et al. used Metropolis et al.’s idea and applied it to search for feasible solutions and converge to an optimal solution, which they termed “simulated annealing” [47,48,49].

Since then, the development of SA algorithms and their applications have generated a new field of study. While annealing is the process of first heating a solid and then cooling it down slowly, in simulated annealing, the temperature is kept variable to simulate this heating process. Specifically, the temperature is initially set high and is then allowed to “cool down” slowly. The initial heating essentially helps to avoid becoming trapped in a local minimum. As the system cools down, its new structure becomes increasingly fixed, thus firmly setting its final properties. In the end, the free energy of the system is minimized, imitating how a minimum is reached during the annealing process, eventually resulting in an optimized solution [50,51]. For more information on SA, the reader is referred to [52,53].

### 2.2. Model Validation

Model performance is primarily evaluated using three statistical measures: mean absolute error (MAE), root mean squared error (*RMSE*), and correlation coefficient (*R*). The value of *R* ranges from 0 to 1; a higher value of *R* (i.e., closer to 1) indicates better performance [54,55,56]. On the contrary, lower values of *RMSE* and *MAE* indicate better performance [57,58,59]. Mathematically, these three measures are defined as

(1) $$MAE=\frac{\sum_{i=1}^{n}\left|p_{i}-v_{i}\right|}{n}$$

(2) $$RMSE\;=\;\sqrt{\sum_{i=1}^{n}\frac{{\left(p_{i}-v_{i}\right)}^{2}}{n}}$$

(3) $$R^{}=\sqrt{\frac{\sum_{i=1}^{n}(p_{i}-q)(v_{i}-\underline{v})}{\sqrt{\sum_{i=1}^{n}(p_{i}-q{)}^{2}}\sum_{i=1}^{n}(v_{i}-\underline{v}{)}^{2}}}$$

where *n* refers to the number of data points; *p_i_* and *q* are the predicted and mean predicted values of the input data, respectively; and *v_i_* and *v* are the individual values and mean values of concentrations of NO_2_ and CO as atmospheric pollutants, respectively.

## 3. Dataset

While air quality data is abundant, large multivariable datasets to develop models are not. In this work, we used data collected between March 2004 and February 2005 in the center of an unnamed, polluted Italian city with heavy traffic, mainly by cars [60,61]; the data is available in open access from the University of California, Irvine (UCI) machine learning repository. While the original dataset contained 9357 records, one analyzer was out of service, and the corresponding data had to be removed. A multisensor device was used to provide hourly averages of the resistivity expressed by the CO-, NO_x_-, O_3_-, and NO_2_-specific metal oxide (MOX) chemiresistors, a nonmetanic hydrocarbon (NMHC)-targeted MOX sensor [60,61]. The multisensor device also contained sensors to capture the temperature as well as the relative and absolute humidity. In the end, the input parameters contained 6941 responses from the eight inputs previously mentioned. In parallel, five conventional fixed stations provided reference concentration estimations for CO (mg/m^3^), NMHC (g/m^3^), benzene (C_6_H_6_) (g/m^3^), NO_x_ (ppb), and NO_2_ (g/m^3^). These results were considered as outputs of the problem, which were recorded hourly by taking averages of the concentration values. While the original dataset had five outputs, we focused on estimating only concentrations of NO_2_ and CO. Table 1 shows the summary statistics of all the variables used in this study.

**Figure 1:**
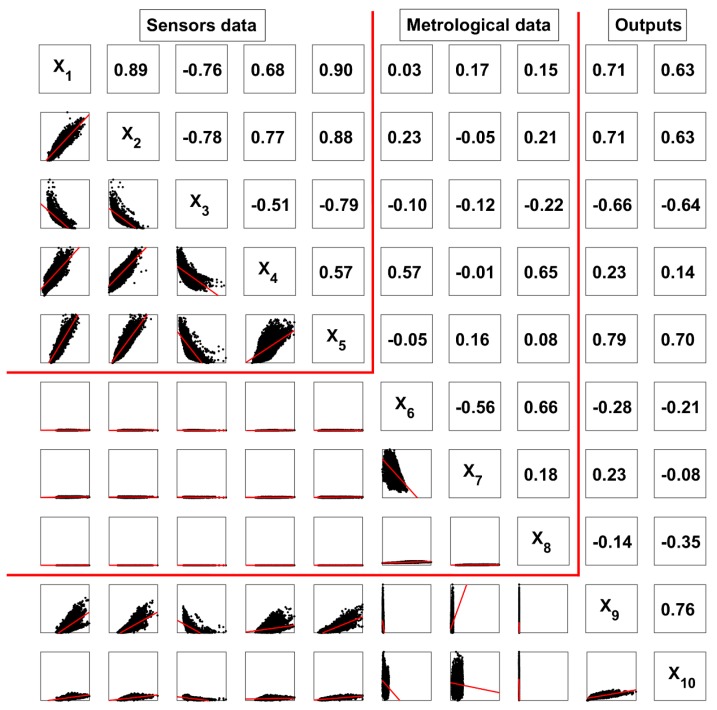
Correlation analysis between sensor resistivities, metrological data, and air quality indexes.

The correlations between the inputs and concentrations of NO_2_ and CO are plotted in Figure 1; both plots and linear correlation coefficients are shown. As Figure 1 clearly shows, some of the variables were significantly correlated. In particular, most of the sensor variables were correlated, although not in a strictly linear fashion. In this work, all variables were included to increase the accuracy of the final models developed.

The training dataset was scaled into the [−1, 1] range, as is common in machine learning, to better follow the non-Gaussian distribution of variables. The scaling process of a variable *x* is expressed by Equation (4), and it involves two parameters, *α* and *β*, shown in Table 1; essentially, *α* is the minimum value of the dataset, and *β* is the maximum value. The same scaling procedure (with the same *α* and *β*) was applied to the testing set as well.

(4) $$x_{scaled}=\frac{2\left(x-\alpha \right)}{\beta -x}-1$$

## 4. Results and Discussion

### 4.1. Optimization Procedure

In this section, the optimization of ANFIS using SA and PSO is detailed. First, we note that there were 250 consequent and antecedent ANFIS parameters to be optimized, corresponding to an eight-dimensional input space. The parameters of ANFIS were generated using C-means clustering. In this work, both input space dimensionality and consuming time were evaluated when choosing the parameters of SA and PSO, especially in terms of population size and maximum number of iterations. Moreover, the maximum number of iterations was chosen as a stopping criterion.

**Figure 2:**
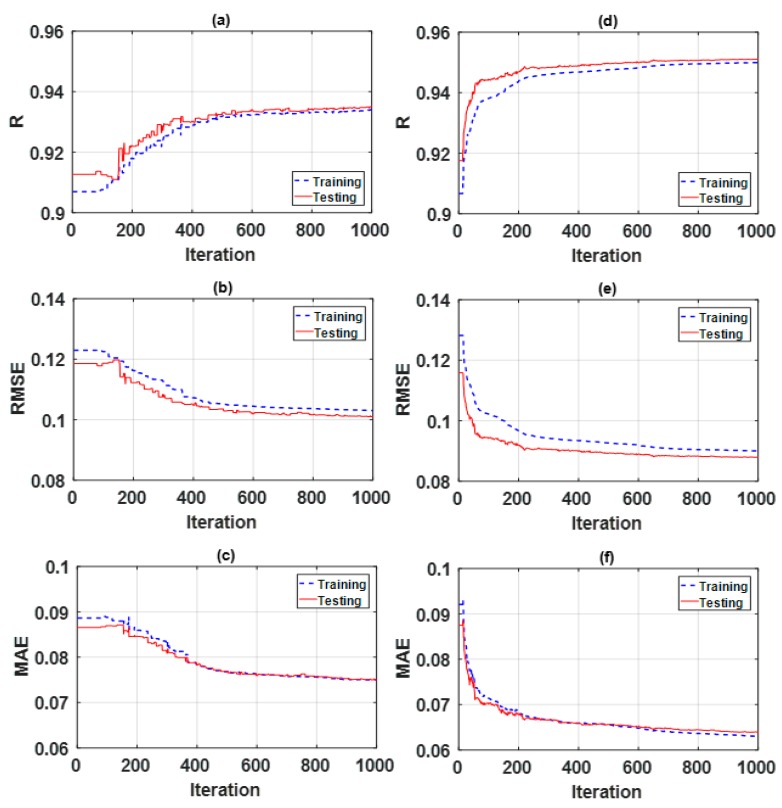
Optimization curves using metaheuristic technique for concentration of NO_2_. Figures for adaptive neuro-fuzzy inference system (ANSIF) optimized by SA (ANFIS-SA): (**a**) correlation coefficient (*R*), (**b**) root mean squared error (*RMSE*), and (**c**) mean absolute error (*MAE*). Figures for ANSIF optimized by PSO (ANFIS-PSO): (**d**) *R*, (**e**) *RMSE*, and (**f**) *MAE*.

**Figure 3:**
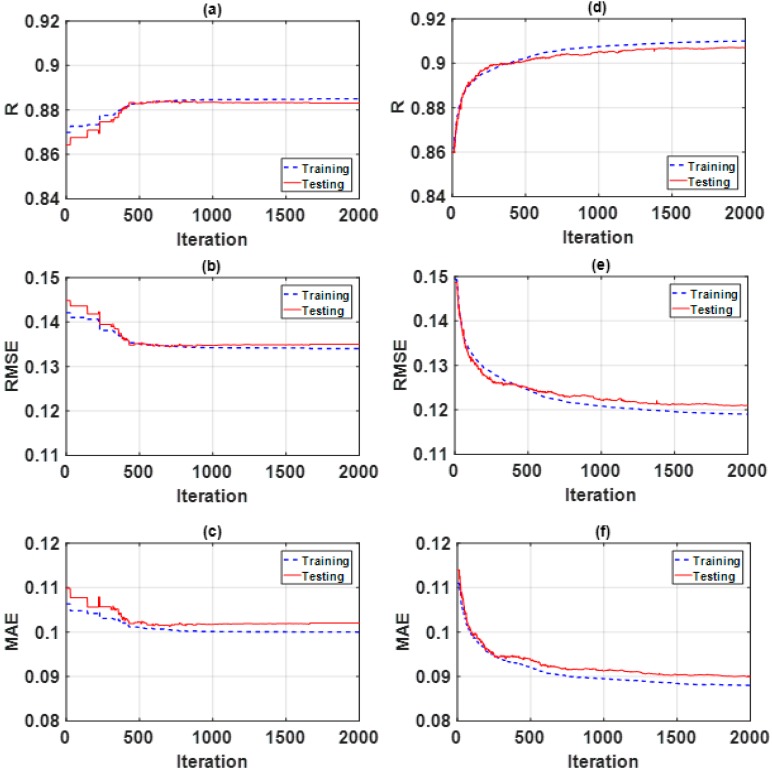
Optimization curves using metaheuristic technique for concentration of CO. Figures for ANFIS-SA: (**a**) *R*, (**b**) *RMSE*, and (**c**) *MAE*. Figures for ANFIS-PSO: (**d**) *R*, (**e**) *RMSE*, and (**f**) *MAE*.

Table 2 and Table 3 show the final parameters selected for SA and PSO, respectively, through a rigorous trial and error process [59,62]. Moreover, optimization curves are presented in Figure 2 for concentration of NO_2_ and in Figure 3 for concentration of CO.

### 4.2. Model Performance

**Figure 4:**
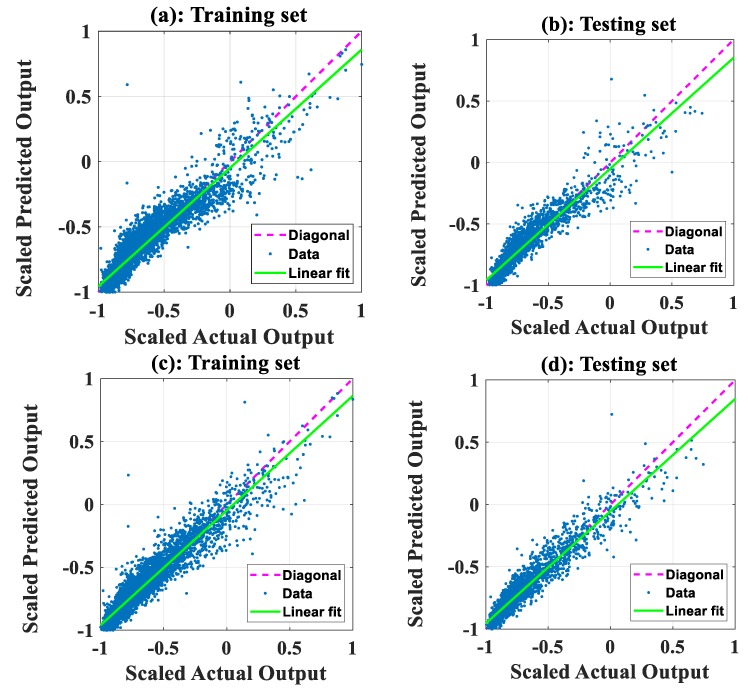
Prediction capability for concentration of NO_2_ in a regression form. Figures using ANFIS-SA for (**a**) training dataset and (**b**) testing dataset. Figures using ANFIS-PSO for (**c**) training dataset and (**d**) testing dataset.

**Figure 5:**
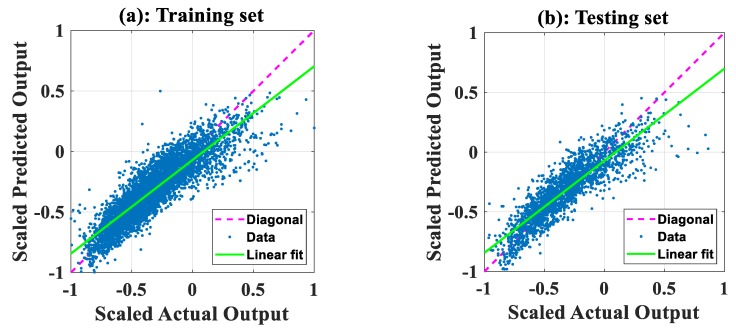

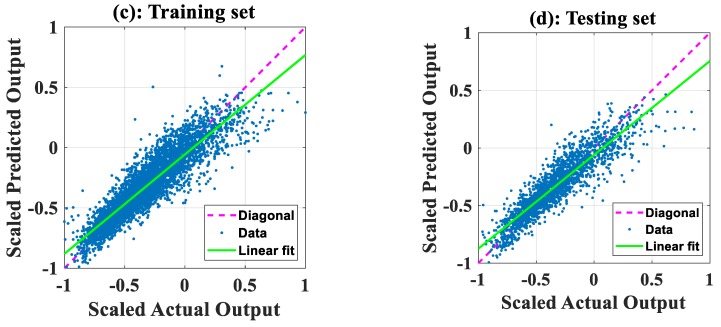
Prediction capability for concentration of CO in a regression form. Figures using ANFIS-SA for (**a**) training dataset and (**b**) testing dataset. Figures using ANFIS-PSO for (**c**) training dataset and (**d**) testing dataset.

The performance of the two models developed is summarized in Table 4. In addition to MAE, *RMSE*, and *R*, a straight line was fitted to predicted vs. actual plots shown in Figure 4 and Figure 5. The slope of the linear fit was then used to measure the angle between the *x*-axis and the linear fit, with angles closer to 45° indicating better performance.

Figure 4a,c shows the prediction capability between the scaled predicted and actual values of NO_2_ concentration on the training set for ANFIS-SA and ANFIS-PSO, respectively. Figure 4b,d shows the same information but applied to the testing set. From the figures and Table 4, we can see that, for the training set, ANFIS-SA and ANFIS-PSO yielded slope angles of 42.23° and 42.34°, respectively. For the testing dataset, ANFIS-SA and ANFIS-PSO produced slope angles of 42.11° and 42.03°, respectively. For NO_2_ concentration, these results suggest that the performance of the two developed models was similar; the three other performance measures suggest similar results.

With regard to the concentration of CO, Figure 5a,c shows the prediction capability of ANFIS-SA and ANFIS-PSO, respectively, using the training dataset. Figure 5b,d shows the same information but applied to the testing set. For the training set, ANFIS-SA and ANFIS-PSO produced slope angles of 37.73° and 39.51°, respectively. For the testing set, ANFIS-SA and ANFIS-PSO generated slope angles of 37.65° and 39.16°, respectively. The ANFIS-PSO therefore performed slightly better than ANFIS-SA. The three other measures support similar conclusions.

**Figure 6:**
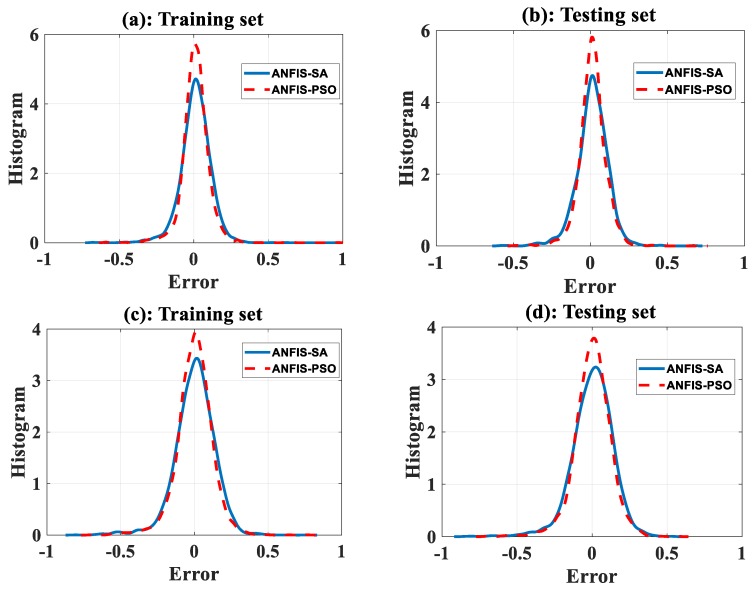
Error analyses for concentration of NO_2_: (**a**) training dataset and (**b**) testing dataset. Error analyses for concentration of CO: (**c**) training dataset and (**d**) testing dataset.

Figure 6a,c shows the histograms of errors of ANFIS-SA and ANFIS-PSO for concentration of NO_2_ using the training and testing datasets, respectively. Figure 6b,d shows the histograms of two models for NO_2_ concentration. We can see that ANFIS-PSO had a higher peak of error concentration around 0 than ANFIS-SA. A similar pattern can be observed for concentration of CO. Moreover, Table 4 shows that the *R* values tended to be higher for ANFIS-PSO, and the *MAE* and *RMSE* values tended to be lower for ANFIS-PSO.

In conclusion, although both models performed well and were statistically significant, ANFIS-PSO was shown to be slightly superior to ANFIS-SA to model CO and NO_2_ concentrations.

### 4.3. Sensitivity Analysis

Predicting air quality is complex as the relationships between the input and target variables are nonlinear. In this section, a sensitivity analysis of the input variables on the predicted results is discussed. In the literature, this type of analysis has been successfully applied to quantify the sensitivity level of input parameters in AI models. For instance, Ly et al. [63] used sensitivity analysis to study the influence of input parameters such as bubble radius, viscosity, and saturation for a problem related to the 3D selective laser sintering process in predicting bubble dissolution time.

The main idea is to exclude one input variable successively from the input space while keeping the others at their median value. Therefore, the method allows us to quantify how sensitive a model is to individual input parameters. Specifically, using the AI prediction model developed previously, a new eight-dimensional input space was constructed based on the probability density distribution of each variable. Here, the value of each input variable was recorded at the following percentiles: 0, 10, 25, 50, 75, 90, and 100. One input variable was then selected, and the model was run seven times, once for each of the seven percentile values. Each time, the other variables were kept at their median value (i.e., 50 percentile). Essentially, the method provided quantitative information on the deviation (i.e., change) of an output when varying the input variables.

In this study, deviation in the output solution, or level of sensitivity $\delta_{i}^{j}$, for the *j*th input variable was expressed as follows:

(5) $$\delta_{i}^{j}=\frac{O_{i}^{j}-O_{ref}^{}}{O_{ref}^{}},$$

where $O_{ref}^{}$ is the output of the configuration of reference, and $O_{i}^{j}$ is the output using *j*th input variable at its *i*th percentile. Finally, the global percentage of sensitivity of each input was computed based on the following equation:

(6) $$\Delta^{j}=\sum_{i=1}^{7}\left|\delta_{i}^{j}\right|$$

**Figure 7:**
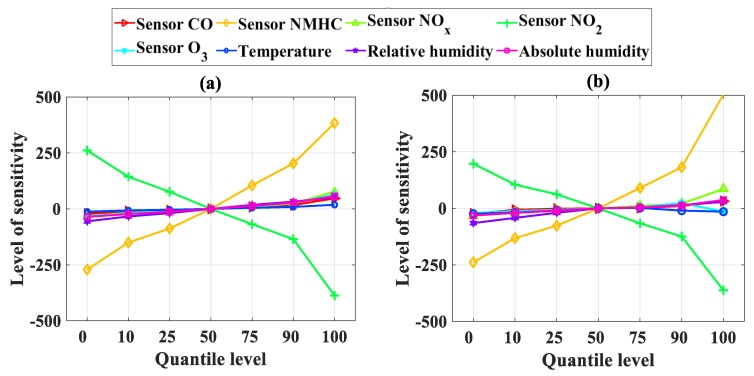
Sensitivity analysis for the concentration of NO_2_: (**a**) using ANFIS-SA and (**b**) using ANFIS-PSO.

**Figure 8:**
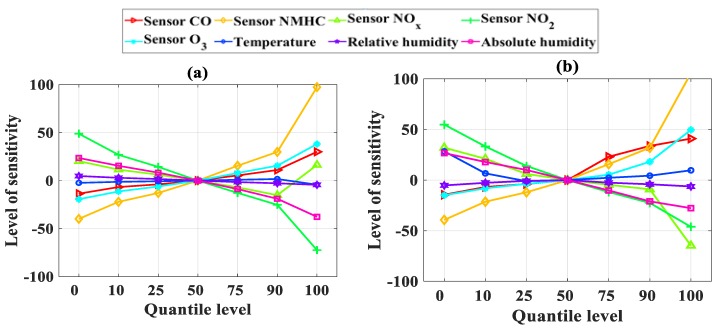
Sensitivity analysis for concentration of CO: (**a**) using ANFIS-SA and (**b**) using ANFIS-PSO.

Table 5 summarizes the values of each input at its seven percentiles, whereas Table 6 summarizes the output solution of the developed AI models corresponding to each percentile. Sensitivity, as a function of the percentile, is plotted in Figure 7 for NO_2_ and in Figure 8 for CO. We can see that, for NO_2_, the input parameters X_2_ (sensor NMHC) and X_4_ (sensor NO_2_) had the most important influence on the predicted results, both for ANFIS-SA and ANFIS-PSO. In addition, the other input parameters had a low impact on the predicted results compared to sensors NMHC and NO_2_ (which was expected as NO_2_ concentration was measured, thus also partly validating the accuracy of the models developed).

In terms of CO concentration, the sensitivity levels of the input parameters fluctuated significantly more; their level of sensitivity can also be consulted in Table 6. Similar to the NO_2_ concentration, NMHC also had the most important impact in terms of sensitivity. For CO, the CO, O_3_, NO_x_, and NO_2_ sensors were also found to have a significant impact. It is also worth noting that the input variables X_6_ (temperature) and X_7_ (relative humidity) had the lowest impact on the predicted results.

In conclusion, from the sensitivity analysis, the NMHC and NO_2_ sensors were the most important parameters in the input space. This means that excluding one of them from the input space would impact the accuracy of the model. It is interesting to notice that using a dataset with 9357 records, De Vito et al. [61] found similar observations.

In their work, to estimate NO_2_ concentration, the best results came from the use of all sensors. In other words, omitting the NMHC or NO_2_ sensors led to lower performance. Interestingly, this was not the case for the CO concentration model. In fact, De Vito et al. [61] found that coupling the CO sensor with NMHC gave the best performance and that including the NO_2_ sensor actually led to lower performance. This phenomenon might be a result of the size of the dataset, with 6941 data points in our study compared with 9357 records in the case of De Vito et al. [61].

**Figure 9:**
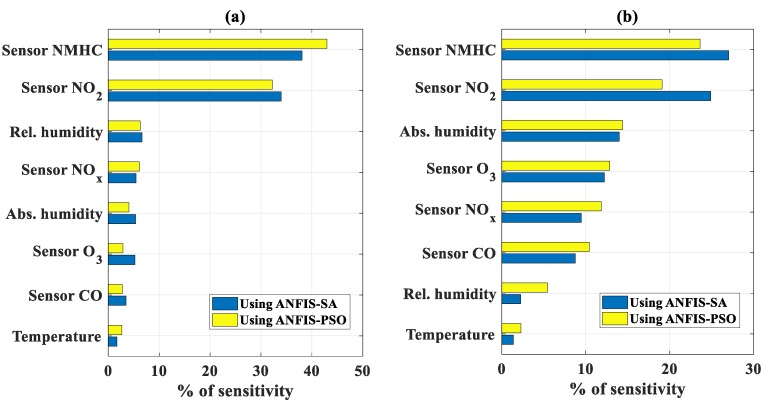
Total percentage of sensitivity for (**a**) concentration of NO_2_ and (**b**) concentration of CO using ANFIS-SA and ANFIS-PSO, respectively.

The total percentage of sensitivity, calculated by summing all levels of sensitivity for each input variables (in absolute values), is presented in Figure 9a for NO_2_ concentration and Figure 9b for CO concentration. The NMHC and NO_2_ sensors appeared as the most important variables to predict both NO_2_ and CO concentrations.

## 5. Conclusions

Predicting air quality accurately is paramount in many cities around the world that are suffering from chronic and severe air pollution problems, notably linked to emissions from fossil fuel-powered transport vehicles. The main goal of this study was to develop an AI model that can reliably predict hourly NO_2_ and CO concentrations from gas multisensor and local weather data. A total of eight input variables were used, consisting of five sensor variables and three weather variables. Moreover, two AI models were trained and tested, namely, ANFIS-PSO and ANFIS-SA.

First, the technical details of the two models and the dataset were introduced and discussed. The results showed that both models performed well and were statistically significant but that ANFIS-PSO performed slightly better. To further investigate the role of each individual input variable in the models developed, a detailed sensitivity analysis was carried out. It was found that the NMHC and NO_2_ sensors particularly affected the sensitivity of both the NO_2_ and CO concentration models. The CO concentration model was shown to be generally more sensitive to all variables. Nonetheless, the three weather variables did not overly affect the accuracy of the model.

Overall, accurately modeling air quality is paramount as the health of millions of people is affected by poor air quality. We have shown that combining multioutput sensor data with advanced AI techniques offers a powerful avenue, especially to model nonlinear processes such as air quality, as was done in this study. Thanks to the collection of new and larger datasets, future work should focus on developing new techniques that can analyze the problem as time series to further improve prediction performance, possibly as done in [64,65,66]. Finally, interested readers are recommended to consider cross-interference, sensitivity, and response time of sensors [67] in AI models developed to predict air quality.

## Figures and Tables

**Table 1:** Dataset parameters and statistical analysis.

Parameters	Sensor CO	Sensor NMHC	Sensor NO_x_	Sensor NO_2_	Sensor O_3_	Temperature	Relative Humidity	Absolute Humidity	C(NO_2_) *	C(CO) **
Role	Input	Input	Input	Input	Input	Input	Input	Input	Output	Output
Notation	X_1_	X_2_	X_3_	X_4_	X_5_	X_6_	X_7_	X_8_	X_9_	X_10_
Min (*α*)	647	390	322	551	221	−1.9	9.2	0.18	2	0.1
Average	1120	959	817	1453	1058	17.8	48.9	0.99	114	2.18
Median	1085	931	786	1457	1006	16.8	49.2	0.95	110	1.90
Max (*β*)	2040	2214	2683	2775	2523	44.6	88.7	2.2	333	11.9
Std	219	264	252	353	407	8.84	17.4	0.40	47	1.44
CV (%)	20	28	31	24	38	50	36	41	42	66

* denotes the concentration of NO_2_; ** denotes the concentration of CO.

**Table 2:** Parameters of simulated annealing (SA) used in this study.

Parameter	NO_2_ Concentration	CO Concentration
Population size	40	60
Maximum number of iterations	1000	2000
Initial temperature	0.1	0.1
Temperature reduction rate	0.99	0.99
Number of neighbors per individual	5	5
Mutation rate	0.5	0.5
Mutation standard deviation	10%	10%

**Table 3:** Parameters of particle swarm optimization (PSO) used in this study.

Parameter	NO_2_ Concentration	CO Concentration
Swarm size	30	50
Maximum number of iterations	1000	2000
Inertia weight	0.4	0.4
Personal learning coefficient	1	1
Global learning coefficient	2	2
Maximum velocity	5	5
Minimum velocity	−5	−5

**Table 4:** Summary information for prediction capability of the scaled data.

Output	Dataset	Model	*R*	*RMSE*	*MAE*	Std Error	Slope
Concentration of NO_2_	Training	ANFIS-SA	0.934	0.103	0.075	0.102	42.23
		ANFIS-PSO	0.950	0.090	0.063	0.089	42.34
	Testing	ANFIS-SA	0.935	0.101	0.075	0.100	42.11
		ANFIS-PSO	0.951	0.088	0.064	0.087	42.03
Concentration of CO	Training	ANFIS-SA	0.885	0.134	0.100	0.134	37.73
		ANFIS-PSO	0.910	0.119	0.088	0.119	39.51
	Testing	ANFIS-SA	0.883	0.135	0.102	0.135	37.65
		ANFIS-PSO	0.907	0.121	0.090	0.121	39.16

**Table 5:** Values of the seven percentiles of each input in the scaled space.

Variable/Percentile	P_0_	P_10_	P_25_	P_50_	P_75_	P_90_	P_100_
Sensor CO	−1.00	−0.68	−0.55	−0.38	−0.13	0.13	1.00
Sensor NMHC	−1.00	−0.74	−0.61	−0.42	−0.19	0.02	1.00
Sensor NO_x_	−1.00	−0.83	−0.73	−0.61	−0.47	−0.31	1.00
Sensor NO_2_	−1.00	−0.64	−0.43	−0.19	0.02	0.22	1.00
Sensor O_3_	−1.00	−0.72	−0.54	−0.32	−0.05	0.22	1.00
Temperature	−1.00	−0.64	−0.44	−0.20	0.10	0.37	1.00
Relative humidity	−1.00	−0.60	−0.32	0.04	0.37	0.64	1.00
Absolute humidity	−1.00	−0.73	−0.50	−0.23	0.06	0.39	1.00

**Table 6:** Summary of level of sensitivity $\delta_{i}^{j}$ of each input at six percentiles. By definition, $\delta_{50}^{j}$ of *j*th input is zero.

Output	Model Used	Variable	Q_0_	Q_10_	Q_25_	Q_75_	Q_90_	Q_100_
Concentration of NO_2_	ANFIS-SA	Sensor CO	−21.37	−10.57	−6.11	8.44	17.36	47.12
		Sensor NMHC	−270.95	−150.42	−87.62	105.13	203.01	383.83
		Sensor NO_x_	−31.77	−17.89	−9.63	10.89	23.76	77.76
		Sensor NO_2_	261.32	143.70	76.46	−68.35	−134.97	−387.09
		Sensor O_3_	−32.66	−18.99	−10.31	13.29	26.12	64.10
		Temperature	−12.33	−6.84	−3.70	4.62	8.77	18.41
		Relative humidity	−55.45	−33.83	−18.95	17.94	32.28	51.37
		Absolute humidity	−35.46	−23.21	−12.45	13.31	28.47	56.79
	ANFIS-PSO	Sensor CO	−23.66	−6.49	−1.72	1.92	10.26	31.96
		Sensor NMHC	−238.61	−131.82	−76.29	90.17	181.71	503.04
		Sensor NO_x_	−31.78	−20.34	−9.95	10.62	21.01	85.62
		Sensor NO_2_	197.17	105.12	62.02	−66.11	−124.34	−362.43
		Sensor O_3_	−20.76	−11.92	−6.43	5.03	23.26	−14.37
		Temperature	−23.93	−18.44	−9.76	1.85	−9.99	−14.60
		Relative humidity	−65.34	−42.17	−19.60	4.16	14.59	28.51
		Absolute humidity	−31.50	−20.21	−10.33	3.67	12.48	36.95
Concentration of CO	ANFIS-SA	Sensor CO	−13.61	−6.73	−3.89	5.38	11.05	30.00
		Sensor NMHC	−39.86	−22.13	−12.89	15.47	29.87	97.39
		Sensor NO_x_	20.32	11.44	6.16	−6.96	−15.19	16.31
		Sensor NO_2_	48.86	26.87	14.30	−12.78	−25.23	−72.37
		Sensor O_3_	−19.42	−11.29	−6.13	7.90	15.53	38.11
		Temperature	−2.34	−1.30	−0.70	0.88	1.67	−4.24
		Relative humidity	4.78	2.92	1.63	−1.55	−2.78	−4.43
		Absolute humidity	23.56	15.42	8.27	−8.84	−18.92	−37.73
	ANFIS-PSO	Sensor CO	−14.49	−6.86	−3.87	23.01	33.73	40.94
		Sensor NMHC	−39.36	−21.38	−12.07	15.71	31.91	105.58
		Sensor NO_x_	32.00	21.01	6.11	−4.66	−9.13	−64.71
		Sensor NO_2_	54.82	33.23	13.90	−11.84	−22.59	−46.19
		Sensor O_3_	−15.02	−8.14	−3.72	5.30	18.09	49.56
		Temperature	28.37	6.63	−1.08	2.28	4.32	9.63
		Relative humidity	−5.28	−2.71	−0.80	−2.57	−4.15	−6.30
		Absolute humidity	26.66	17.85	9.96	−10.23	−21.02	−27.74

## References

[1] Derrible S., Saneinejad S., Sugar L., Kennedy C. (2010). Macroscopic Model of Greenhouse Gas Emissions for Municipalities. Transp. Res. Rec..

[2] Anenberg S.C., Miller J., Minjares R., Du L., Henze D.K., Lacey F., Malley C.S., Emberson L., Franco V., Klimont Z. (2017). Impacts and mitigation of excess diesel-related NO_x_ emissions in 11 major vehicle markets. Nature.

[3] Streets D.G., Waldhoff S.T. (2000). Present and future emissions of air pollutants in China: SO_2_, NO_x_, and CO. Atmos. Environ..

[4] Miller K.A., Siscovick D.S., Sheppard L., Shepherd K., Sullivan J.H., Anderson G.L., Kaufman J.D. (2007). Long-term exposure to air pollution and incidence of cardiovascular events in women. N. Engl. J. Med..

[5] Raaschou-Nielsen O., Andersen Z.J., Hvidberg M., Jensen S.S., Ketzel M., Sørensen M., Loft S., Overvad K., Tjønneland A. (2011). Lung cancer incidence and long-term exposure to air pollution from traffic. Environ. Health Perspect..

[6] Raaschou-Nielsen O., Andersen Z.J., Beelen R., Samoli E., Stafoggia M., Weinmayr G., Hoffmann B., Fischer P., Nieuwenhuijsen M.J., Brunekreef B. (2013). Air pollution and lung cancer incidence in 17 European cohorts: Prospective analyses from the European Study of Cohorts for Air Pollution Effects (ESCAPE). Lancet Oncol..

[7] Beelen R., Hoek G., Van Den Brandt P.A., Goldbohm R.A., Fischer P., Schouten L.J., Armstrong B., Brunekreef B. (2008). Long-term exposure to traffic-related air pollution and lung cancer risk. Epidemiology.

[8] Brunekreef B., Beelen R., Hoek G., Schouten L., Bausch-Goldbohm S., Fischer P., Armstrong B., Hughes E., Jerrett M., van den Brandt P. (2009). Effects of long-term exposure to traffic-related air pollution on respiratory and cardiovascular mortality in the Netherlands: The NLCS-AIR study. Res. Rep. Health Eff. Inst..

[9] Hystad P., Demers P.A., Johnson K.C., Carpiano R.M., Brauer M. (2013). Long-term residential exposure to air pollution and lung cancer risk. Epidemiology.

[10] Götschi T., Heinrich J., Sunyer J., Künzli N. (2008). Long-term effects of ambient air pollution on lung function: A review. Epidemiology.

[11] Mohareb E., Derrible S., Peiravian F. (2016). Intersections of Jane Jacobs’ Conditions for Diversity and Low-Carbon Urban Systems: A Look at Four Global Cities. J. Urban Plan. Dev..

[12] Cimorelli A.J., Perry S.G., Venkatram A., Weil J.C., Paine R.J., Wilson R.B., Lee R.F., Peters W.D., Brode R.W. (2005). AERMOD: A dispersion model for industrial source applications. Part I: General model formulation and boundary layer characterization. J. Appl. Meteorol..

[13] Leelőssy Á., Molnár F., Izsák F., Havasi Á., Lagzi I., Mészáros R. (2014). Dispersion modeling of air pollutants in the atmosphere: A review. Cent. Eur. J. Geosci..

[14] Samson P.J. (1988). Atmospheric Transport and Dispersion of Air Pollutants Associated with Vehicular Emissions.

[15] Abiye O.E., Sunmonu L.A., Ajao A.I., Akinola O.E., Ayoola M.A., Jegede O.O. (2016). Atmospheric dispersion modeling of uncontrolled gaseous pollutants (SO_2_ and NO_x_) emission from a scrap-iron recycling factory in Ile-Ife, Southwest Nigeria. Cogent Environ. Sci..

[16] Kota S.H., Ying Q., Zhang Y. (2013). Simulating near-road reactive dispersion of gaseous air pollutants using a three-dimensional Eulerian model. Sci. Total Environ..

[17] Goyal P., Jaiswal N. (2010). Effects of meteorological parameters on RSPM concentration in urban Delhi. Int. J. Environ. Waste Manag..

[18] Nigam S., Nigam R., Kulshrestha M., Mittal S.K. (2010). Carbon monoxide modeling studies: A review. Environ. Rev..

[19] Paraschiv D., Tudor C., Petrariu R. (2015). The Textile Industry and Sustainable Development: A Holt–Winters Forecasting Investigation for the Eastern European Area. Sustainability.

[20] Žabkar R., Honzak L., Skok G., Forkel R., Rakovec J., Ceglar A., Žagar N. (2015). Evaluation of the high resolution WRF-Chem (v3.4.1) air quality forecast and its comparison with statistical ozone predictions. Geosci. Model Dev..

[21] Garner G.G., Thompson A.M. (2011). The Value of Air Quality Forecasting in the Mid-Atlantic Region. Weather Clim. Soc..

[22] Abdullah A.B.M., Mitchell D., Pavur R. (2009). An overview of forecast models evaluation for monitoring air quality management in the State of Texas, USA. Manag. Environ. Qual. Int. J..

[23] Russo A., Lind P.G., Raischel F., Trigo R., Mendes M. (2014). Daily pollution forecast using optimal meteorological data at synoptic and local scales. arXiv.

[24] Peng J., Huang Y., Liu T., Jiang L., Xu Z., Xing W., Feng X., De Maeyer P. (2019). Atmospheric nitrogen pollution in urban agglomeration and its impact on alpine lake-case study of Tianchi Lake. Sci. Total Environ..

[25] Sharma R., Kumar R., Sharma D.K., Son L.H., Priyadarshini I., Pham B.T., Tien Bui D., Rai S. (2019). Inferring air pollution from air quality index by different geographical areas: Case study in India. Air Qual. Atmos. Health.

[26] Shi J.P., Harrison R.M. (1997). Regression modelling of hourly NO_x_ and NO_2_ concentrations in urban air in London. Atmos. Environ..

[27] Lee D., Derrible S., Pereira F.C. (2018). Comparison of Four Types of Artificial Neural Network and a Multinomial Logit Model for Travel Mode Choice Modeling. Transp. Res. Rec..

[28] Thanh T.T.M., Ly H.-B., Pham B.T., Ha-Minh C., Dao D.V., Benboudjema F., Derrible S., Huynh D.V.K., Tang A.M. (2019). A Possibility of AI Application on Mode-choice Prediction of Transport Users in Hanoi. CIGOS 2019, Innovation for Sustainable Infrastructure.

[29] Lee D., Derrible S. (2019). Predicting Residential Water Consumption: Modeling Techniques and Data Perspectives. J. Water Resour. Plan. Manag..

[30] Le T.-T., Pham B.T., Ly H.-B., Shirzadi A., Le L.M., Ha-Minh C., Dao D.V., Benboudjema F., Derrible S., Huynh D.V.K., Tang A.M. (2019). Development of 48-hour Precipitation Forecasting Model using Nonlinear Autoregressive Neural Network. CIGOS 2019, Innovation for Sustainable Infrastructure.

[31] Brunelli U., Piazza V., Pignato L., Sorbello F., Vitabile S., Apolloni B., Marinaro M., Nicosia G., Tagliaferri R. (2006). Hourly Forecasting of SO_2_ Pollutant Concentration Using an Elman Neural Network. Neural Nets.

[32] Comrie A.C. (1997). Comparing Neural Networks and Regression Models for Ozone Forecasting. J. Air Waste Manag. Assoc..

[33] Gardner M.W., Dorling S.R. (1999). Neural network modelling and prediction of hourly NO_x_ and NO_2_ concentrations in urban air in London. Atmos. Environ..

[34] Jang J.-S.R. (1997). Neuro-Fuzzy and Soft Computing: A Computational Approach to Learning and Machine Intelligence.

[35] Bilgehan M. (2011). Comparison of ANFIS and NN models—With a study in critical buckling load estimation. Appl. Soft Comput..

[36] Scherer R., Scherer R. (2012). Takagi-Sugeno Fuzzy Systems. Multiple Fuzzy Classification Systems.

[37] Dao D.V., Ly H.-B., Trinh S.H., Le T.-T., Pham B.T. (2019). Artificial Intelligence Approaches for Prediction of Compressive Strength of Geopolymer Concrete. Materials.

[38] Termeh S.V.R., Khosravi K., Sartaj M., Keesstra S.D., Tsai F.T.-C., Dijksma R., Pham B.T. (2019). Optimization of an adaptive neuro-fuzzy inference system for groundwater potential mapping. Hydrogeol. J..

[39] Kennedy J., Eberhart R. Particle swarm optimization. Proceedings of the ICNN’95—International Conference on Neural Networks.

[40] Perera R., Arteaga A., Diego A. (2010). De Artificial intelligence techniques for prediction of the capacity of RC beams strengthened in shear with external FRP reinforcement. Compos. Struct..

[41] Sehgal V., Sahay R.R., Chatterjee C. (2014). Effect of Utilization of Discrete Wavelet Components on Flood Forecasting Performance of Wavelet Based ANFIS Models. Water Resour. Manag..

[42] Chen W., Panahi M., Pourghasemi H.R. (2017). Performance evaluation of GIS-based new ensemble data mining techniques of adaptive neuro-fuzzy inference system (ANFIS) with genetic algorithm (GA), differential evolution (DE), and particle swarm optimization (PSO) for landslide spatial modelling. CATENA.

[43] Shi Y., Eberhart R.C. Fuzzy adaptive particle swarm optimization. Proceedings of the 2001 Congress on Evolutionary Computation (IEEE Cat. No. 01TH8546).

[44] Poli R., Kennedy J., Blackwell T. (2007). Particle swarm optimization. Swarm Intell..

[45] Dao D.V., Trinh S.H., Ly H.-B., Pham B.T. (2019). Prediction of Compressive Strength of Geopolymer Concrete Using Entirely Steel Slag Aggregates: Novel Hybrid Artificial Intelligence Approaches. Appl. Sci..

[46] Metropolis N., Rosenbluth A.W., Rosenbluth M.N., Teller A.H., Teller E. (1953). Equation of state calculations by fast computing machines. J. Chem. Phys..

[47] Aarts E., Korst J., Michiels W., Burke E.K., Kendall G. (2005). Simulated Annealing. Search Methodologies: Introductory Tutorials in Optimization and Decision Support Techniques.

[48] Vidal R.V.V. (1993). Lecture Notes in Economics and Mathematical Systems. Applied Simulated Annealing.

[49] Aguiar e Oliveira Junior H., Ingber L., Petraglia A., Rembold Petraglia M., Augusta Soares Machado M., Aguiar e Oliveira Junior H., Ingber L., Petraglia A., Rembold Petraglia M., Augusta Soares Machado M. (2012). Metaheuristic Methods. Stochastic Global Optimization and Its Applications with Fuzzy Adaptive Simulated Annealing.

[50] Pham D., Karaboga D. (2000). Intelligent Optimisation Techniques—Genetic Algorithms, Tabu Search, Simulated Annealing and Neural Networks.

[51] Dréo J., Siarry P., Pétrowski A., Taillard E. (2006). Metaheuristics for Hard Optimization.

[52] Romary T., de Fouquet C., Malherbe L. (2011). Sampling design for air quality measurement surveys: An optimization approach. Atmos. Environ..

[53] Vincent F.Y., Redi A.P., Hidayat Y.A., Wibowo O.J. (2017). A simulated annealing heuristic for the hybrid vehicle routing problem. Appl. Soft Comput..

[54] Pham B.T., Nguyen M.D., Dao D.V., Prakash I., Ly H.-B., Le T.-T., Ho L.S., Nguyen K.T., Ngo T.Q., Hoang V. (2019). Development of artificial intelligence models for the prediction of Compression Coefficient of soil: An application of Monte Carlo sensitivity analysis. Sci. Total Environ..

[55] Le L.M., Ly H.-B., Pham B.T., Le V.M., Pham T.A., Nguyen D.-H., Tran X.-T., Le T.-T. (2019). Hybrid Artificial Intelligence Approaches for Predicting Buckling Damage of Steel Columns Under Axial Compression. Materials.

[56] Pham B.T., Nguyen M.D., Ly H.-B., Pham T.A., Hoang V., Van Le H., Le T.-T., Nguyen H.Q., Bui G.L., Ha-Minh C., Dao D.V., Benboudjema F., Derrible S., Huynh D.V.K., Tang A.M. (2019). Development of Artificial Neural Networks for Prediction of Compression Coefficient of Soft Soil. CIGOS 2019, Innovation for Sustainable Infrastructure.

[57] Ly H.-B., Desceliers C., Le L.M., Le T.-T., Pham B.T., Nguyen-Ngoc L., Doan V.T., Le M. (2019). Quantification of Uncertainties on the Critical Buckling Load of Columns under Axial Compression with Uncertain Random Materials. Materials.

[58] Nguyen H.-L., Le T.-H., Pham C.-T., Le T.-T., Ho L.S., Le V.M., Pham B.T., Ly H.-B. (2019). Development of Hybrid Artificial Intelligence Approaches and a Support Vector Machine Algorithm for Predicting the Marshall Parameters of Stone Matrix Asphalt. Appl. Sci..

[59] Ly H.-B., Pham B.T., Dao D.V., Le V.M., Le L.M., Le T.-T. (2019). Improvement of ANFIS Model for Prediction of Compressive Strength of Manufactured Sand Concrete. Appl. Sci..

[60] De Vito S., Massera E., Piga M., Martinotto L., Di Francia G. (2008). On field calibration of an electronic nose for benzene estimation in an urban pollution monitoring scenario. Sens. Actuators B Chem..

[61] De Vito S., Piga M., Martinotto L., Di Francia G. (2009). CO, NO_2_ and NO_x_ urban pollution monitoring with on-field calibrated electronic nose by automatic bayesian regularization. Sens. Actuators B Chem..

[62] Ly H.-B., Le L.M., Duong H.T., Nguyen T.C., Pham T.A., Le T.-T., Le V.M., Nguyen-Ngoc L., Pham B.T. (2019). Hybrid Artificial Intelligence Approaches for Predicting Critical Buckling Load of Structural Members under Compression Considering the Influence of Initial Geometric Imperfections. Appl. Sci..

[63] Ly H.-B., Monteiro E., Le T.-T., Le V.M., Dal M., Regnier G., Pham B.T. (2019). Prediction and Sensitivity Analysis of Bubble Dissolution Time in 3D Selective Laser Sintering Using Ensemble Decision Trees. Materials.

[64] Dell’Acqua F., Iannelli G., Torres M., Martina M. (2018). A Novel Strategy for Very-Large-Scale Cash-Crop Mapping in the Context of Weather-Related Risk Assessment, Combining Global Satellite Multispectral Datasets, Environmental Constraints, and In Situ Acquisition of Geospatial Data. Sensors.

[65] Alpers W., Dagestad K.-F., Wong W.K., Chan P.W. A winter monsoon front over the South China Sea studied by multi-sensor satellite data, weather radar data, and a numerical model. Proceedings of the 2012 IEEE International Geoscience and Remote Sensing Symposium.

[66] Halem M., Most N., Tilmes C.A., Stewart K., Yesha Y., Chapman D., Nguyen P., Sensing R. (2008). Service-oriented atmospheric radiances (SOAR): Gridding and analysis services for multisensor aqua IR radiance data for climate studies. IEEE Trans. Geosci. Remote Sens..

[67] Beliatis M.J., Rozanski L.J., Jayawardena K.I., Rhodes R., Anguita J.V., Mills C.A., Silva S.R.P. (2015). Hybrid and Nano-composite Carbon Sensing Platforms. Carbon for Sensing Devices.

